# Amplicon sequencing data profiling of bacterial community connected with the rhizospheric soil from sunflower plants

**DOI:** 10.1016/j.dib.2022.108207

**Published:** 2022-04-26

**Authors:** Olubukola Oluranti Babalola, Blessing Chidinma Nwachukwu, Ayansina Segun Ayangbenro

**Affiliations:** Food Security and Safety Focus Area, Faculty of Natural and Agricultural Sciences, North-West University, Private Mail Bag X2046, South Africa

**Keywords:** 16S rRNA gene, Illumina MiSeq system, Microbial community, Operational taxonomic units

## Abstract

This article presents dataset on the bacterial community structure associated with rhizospheric sunflower soils from Lichtenburg, South Africa. The Illumina Miseq sequencing of 16S rRNA gene amplicon unveiled the bacterial community diversities as well as generated metagenomic library from sunflower rhizospheric soils to understand the diversities and distribution. The metagenome contained a total of 41,471 sequences with 45,975 operational taxonomic units (OTUs). Metagenome sequence information is obtainable at NCBI under the Bioproject assigned accession number PRJNA782103. Taxonomic hits distribution from USEARCH analysis at phylum level classification of BN_E discovered predominantly Actinobacteria (33.89%), followed by Proteobacteria (35.45%), Firmicutes (6.45%), Planctomycetes (4.12%), Chloroflexi (4.28%) and Gemmatimonadetes (2.40%). Also, USEARCH assisted analysis of BN_F sample also detected the prevalence of Actinobacteria (45.92%), Proteobacteria (23.23%), Firmicutes (3.84%), Planctomycetes (6.46%), Chloroflexi (4.94%) and Gemmatimonadetes (1.99%), which take part in vital ecological functions and biogeochemical activities needed for plant growth and health.

## Specifications Table

**Table:** 

Subject	Microbiology
Specific subject area	Bacteriology, Next Generation Sequencing (NGS)
Type of data	Amplicon data (Text file and Figures)
How data was acquired	16S rRNA metagenome sequencing was performed on Illumina MiSeq platform (www.illumina.com), and OTU clustering analysis was conducted using USEARCH.
Data format	Raw data (FASTQ)
Parameters for data collection	Sunflower rhizospheric soils were collected, and bacterial diversity were analyzed from the extracted DNA using commercial DNA isolation kit (Zymo® isolation kit, Zymo Research, USA).
Description of data collection	Sequence reads were processed through USEARCH (https://drive5.com/usearch) and taxonomy were assigned using Ribosomal Database Project's (http://rdp.cme.msu.edu/index.jsp) 16 s database v16 database.
Data source location	Sunflower rhizospheric soils were collected from Lichtenburg (S 26°.4`31.398`` E25°.5`84.4286), North West Province, South Africa.
Data accessibility	The raw metagenomic DNA sequences has been deposited into NCBI repository with Bioproject ID: PRJNA782103. https://www.ncbi.nlm.nih.gov/bioproject/PRJNA782103. The Operational Taxonomic Units (OTUs) tables have been deposited into Mendeley Data repository “Operational Taxonomic Units of metagenomes from rhizospheric soils of sunflower plant” with DOI: 10.17632/nprdypz7c4.1. https://data.mendeley.com/datasets/nprdypz7c4/1

## Value of the Data

• The data presented in this study are important because exploiting sunflower-associated rhizosphere bacterial community structure with active novel genes and functions have promising potentials in promoting sustainable agriculture.
• The data information of culturable and unculturable bacterial population evaluated can be used by researchers and farmers for improved production of agricultural crops, such as sunflower.
• The datasets can be employed to estimate the roles of various bacterial species in maintaining the rhizosphere, which are important for plant growth and in solving the problem of food security.

## 1 Data Description

The dataset contains a raw metagenomic sequence obtained using amplicon sequencing of the sunflower rhizosphere bacterial community. The data files in FASTQ format can be found on National Center for Biotechnology Information (NCBI) portal with SRA accession number Bioproject ID: PRJNA782103.

**Fig. 1:**
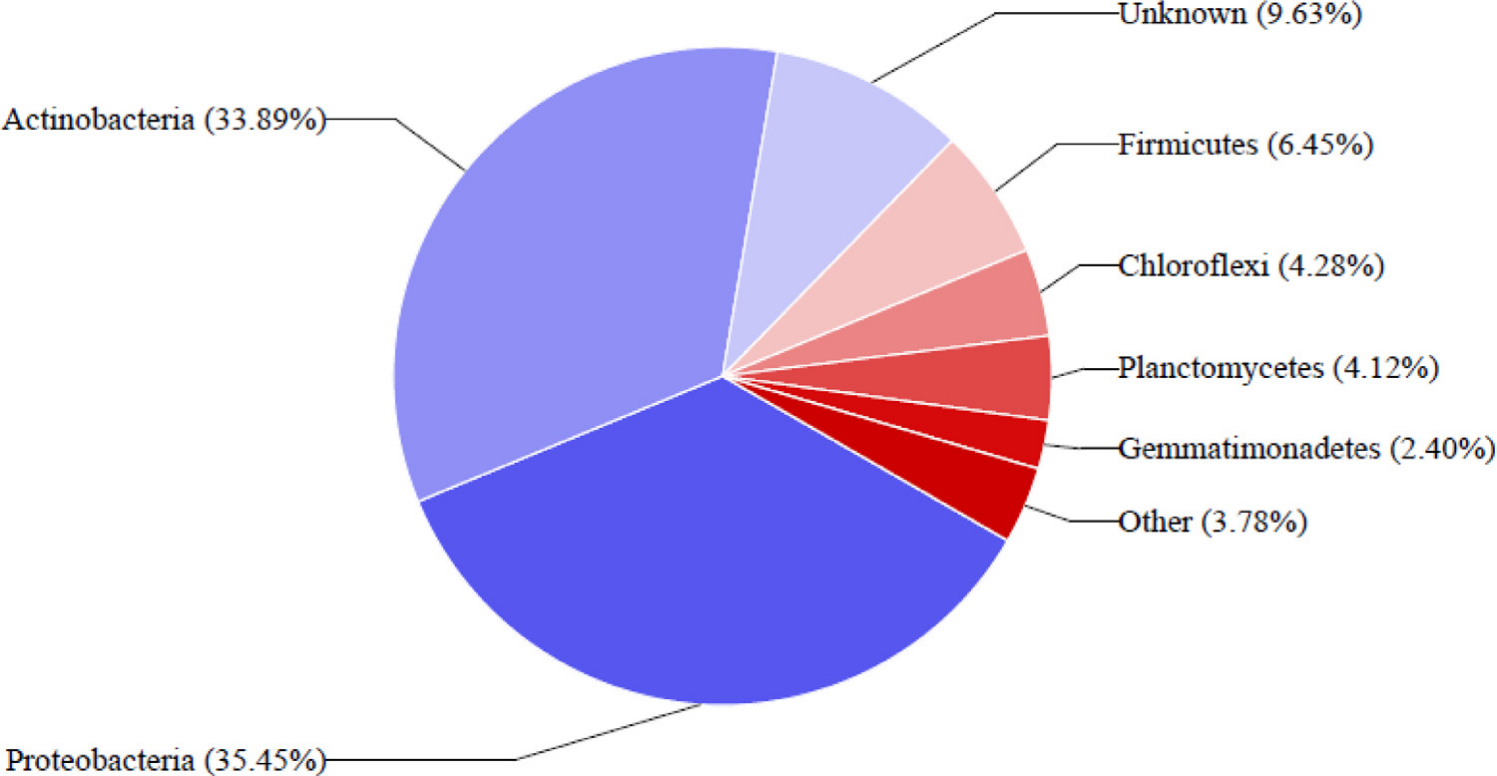
Percentage of key bacterial composition from sunflower rhizosphere soil samples (BN_E).

**Fig. 2:**
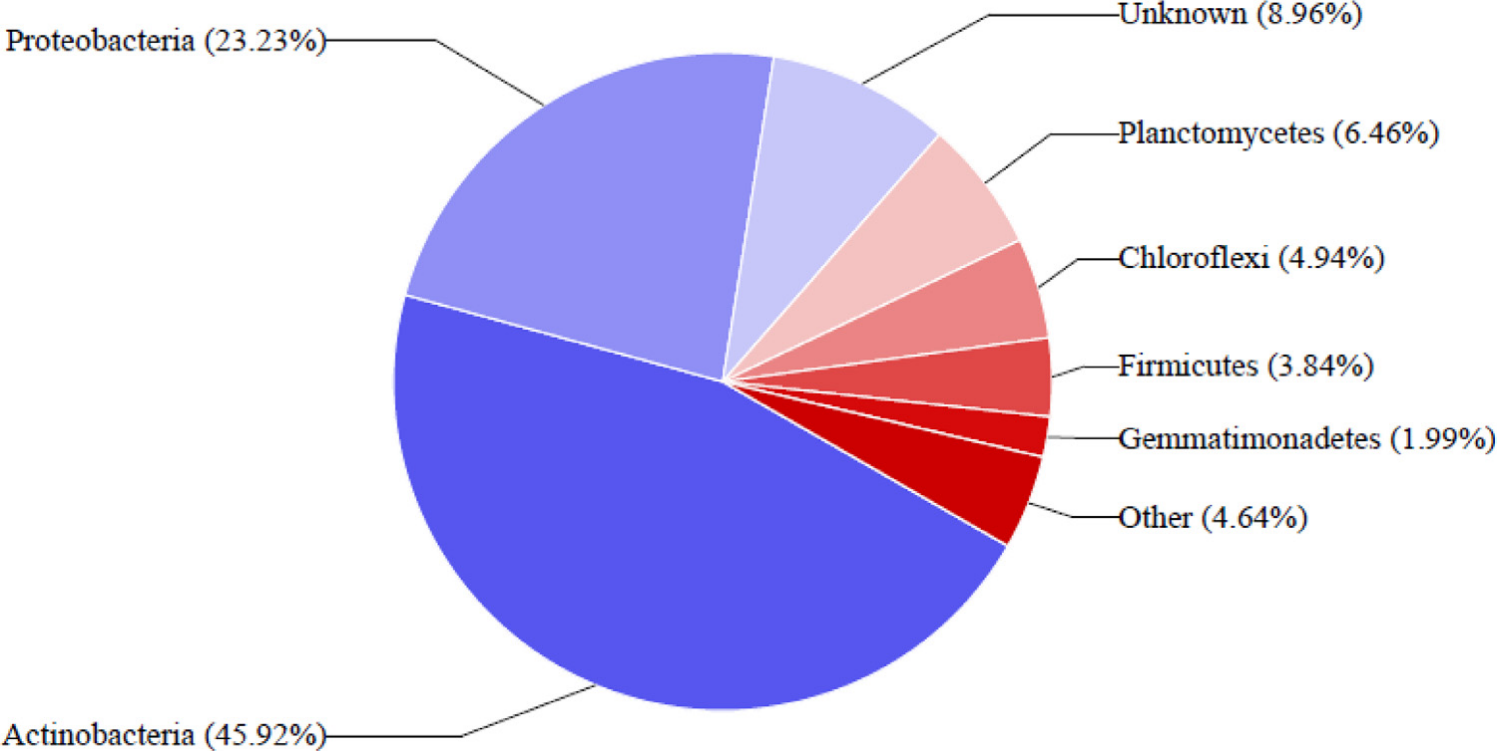
Percentage of key bacterial communities identified from sunflower rhizosphere soil samples (BN_F).

A total of 26,854 (BN_E) and 20,617 (BN_F) reads were analyzed, and 25,842 (BN_E) and 20,133 (BN_F) operational taxonomic units (OTUs) were classified in order to reveal the diversity of bacterial community. All the resulting OTUs were then classified into phyla, classes, orders, families and genera. USEARCH analysis at phylum level classification discovered preponderantly Actinobacteria followed by Proteobacteria, Firmicutes, Planctomycetes, Chloroflexi and Gemmatimonadetes. The data are obtainable in Fig. 1, Fig. 2, respectively. The categorized OTUs represent distinct classes dominated by Actinobacteria, Alphaproteobacteria, Gammaproteobacteria, Bacilli, Thermoleophilia Rubrobacteria Betaproteobacteria and Phycisphaerae. At orders level Actinomycetales, Pseudomonadales, Caulobacterales, Bacillales, Rhizobiales, Caulobacterales, Rhodospirillales, and Rubrobacterales had higher OTUs. Families with the highest level of dominance were Caulobacteraceae Bacillaceae, Pseudomonadaceae, Rubrobacteraceae, Geodermatophilaceae, Micrococcaceae, Rubrobacteraceae, Geodermatophilaceae, Nocardioidaceae, Bradyrhizobiaceae and Acetobacteraceae. The analysis classified 10 most dominant genera were Pseudoalteromonas, Arthrobacter, Rubrobacter, Balneimonas, Bacillus, Nocardioides, Acinetobacter, Rubrobacter Pseudomonas, Brevundimonas and Modestobacter. The 16S rRNA amplicon sequence information of the six most dominant OTUs in the bacterial species estimated in the rhizospheric soil of sunflower are shown in Table 1.

**Table 1:** Identities of the six most dominant OTUs in the bacterial communities from sunflower rhizosphere soil.

	Taxa (Read count and % of abundance)	
Relative abundance order	BN_E	BN_F
1	Acinetobacter_johnsonii (1482.0; 5.57%)	Rubrobacter_ (1030.0; 5.06%)
2	Rubrobacter_ (1071.0; 4.03%)	Balneimonas_ (433.0; 2.13%)
3	Acinetobacter_guillouiae (720.0; 2.71%)	Modestobacter_ (255.0; 1.25%)
4	Brevundimonas_poindexterae (639.0; 2.40%)	Arthrobacter_pascens (222.0; 1.09%)
5	Bacillus_cereus (575.0; 2.16%)	Arthrobacter_ (158.0; 0.78%)
6	Pseudomonas_fragi (396.0; 1.49%)	Solirubrobacter_ (150.0; 0.74%)

## 2 Experimental Design, Materials and Methods

In this dataset, rhizosphere soil samples were collected from sunflower rhizosphere soils in Lichtenburg, South Africa (26.431398S: 25.5844286E) in March, 2020 at two different sites (BN_E and BN_F) and the metagenomic DNA extraction was performed using the Zymo® DNA isolation kit (Zymo Research, USA) following the manufacturer's instruction [1]. Metagenomic DNA purity and concentration were ascertained using a NanoDrop Lite Spectrophotometer (Thermo Fischer Scientific, CA, USA). Sequencing was performed using the Illumina Miseq platform at Inqaba Biotechnical Industries (Pty) Ltd, Pretoria, South Africa. The initial metagenomic DNA quality was evaluated using Qubit® dsDNA HS Assay Kit (Life Technologies) The 16S rRNA library preparation was done using 16S rRNA gene universal primers (Forward Primer: ACTCCTACGGGAGGCAGCAG) and (Reverse Primer: GGACTACHVGGGTWTCTAAT) with standard Illumina adapters and barcodes [2]. In contrast, the Ampure XP beads were used to further purify the Amplicons [3]. The barcoded libraries were determined by Agilent DNA 1000 Bioanalyser and Qubit DNA BR reagent assay was used for quantification. The quantified libraries were pooled and sequenced using MiSeq [4]. Reads were processed through USEARCH (https://drive5.com/usearch) [4] and taxonomic information was determined based on the Ribosomal Database Project's (http://rdp.cme.msu.edu/index.jsp) 16 s database v16 database [5]. The seed sequence for each cluster was arranged by length and clustered with a 3% divergence cut-off to create centroid clusters. Operational Taxonomic Units (OTUs) contributing less than 1% in size of the total data was excluded [6].

## Ethics Approval and Consent to Participate

Not applicable.

## CRediT authorship contribution statement

**Olubukola Oluranti Babalola:** Funding acquisition, Resources, Supervision. **Blessing Chidinma Nwachukwu:** Conceptualization, Data curation, Formal analysis, Investigation, Methodology, Writing – original draft, Writing – review & editing. **Ayansina Segun Ayangbenro:** Conceptualization, Project administration, Writing – review & editing.

## Declaration of Competing Interest

The authors declare that they have no conflict of interest, either financial or commercial wise.

## Data Availability

Functional and microbial diversity study of sunflower rhizosphere from Lichtenburg (Original data) (National Center for Biotechnology Information) with SRA accession number Bioproject ID: PRJNA782103. Operational Taxonomic Units of metagenomes from rhizospheric soils of sunflower plant (Operational Taxonomic Units- OTUs tables) (Mendeley Data) with DOI: 10.17632/nprdypz7c4.1.

## Appendix Supplementary materials

Image, application 1

Image, application 2

Image, application 3

Image, application 4

Image, application 5

Image, application 6

Image, application 7

Image, application 8

Image, application 9

Image, application 10
